# Identification of a novel miR‐21‐3p/TGF‐β signaling‐driven immune escape via the MHC class I/biglycan axis in tumor cells

**DOI:** 10.1002/ctm2.306

**Published:** 2021-03-24

**Authors:** Karthikeyan Subbarayan, Chiara Massa, Maria‐Filothei Lazaridou, Kamatchi Ulagappan, Barbara Seliger

**Affiliations:** ^1^ Institute of Medical Immunology Martin Luther University Halle‐Wittenberg Halle Germany; ^2^ Fraunhofer Institute for Cell Therapy and Immunology Leipzig Germany

Dear Editor,

For the first time, a miR‐21‐3p‐mediated downregulation of MHC class I surface antigens was shown in different model systems, which is linked to the expression of the extracellular matrix (ECM) constituent biglycan (BGN) and transforming growth factor (TGF)‐β signaling in HER‐2/neu‐positive (HER‐2/neu^+^) cells. HER‐2/neu transformation induces the expression of miR‐21‐3p, which interferes with the expression of immune‐modulatory molecules, thereby accelerating immune suppression and reducing tumor immunogenicity.

The oncogenic miR‐21 is overexpressed in many cancers associated with altered growth characteristics leading to tumor progression and reduced patients’ survival.^1^, ^2^ Recently, we have shown that HER‐2/neu transformation resulted in a downregulation of BGN, causing a reduced MHC class I surface expression.^3^ However, a relationship between miR‐21‐3p overexpression, enhanced TGF‐β signaling, impaired BGN, and MHC class I expression in HER‐2/neu^+^ cells has not yet been investigated.

Using murine in vitro models of HER‐2/neu transformation and human tumor cells with a distinct HER‐2/neu status, the role of miR‐21 in the HER‐2/neu‐mediated downregulation of the MHC class I surface antigens, which is accompanied by a reduced BGN expression and increased TGF‐β signaling, was analyzed. The expression of MHC class I antigen processing machinery (APM) and TGF‐β pathway components, BGN and miR‐21‐3p, were determined by qPCR using primers listed in Table S1, Western blot and surface expression by flow cytometry. MiR‐21‐3p targets were identified and validated by binding miR‐21‐3p to the respective 3′ untranslated region (UTR). The function of miR‐21‐3p was assessed upon its overexpression and inhibition. CD107a degranulation assay was used to determine the NK cell activity upon co‐culture of miR‐21‐3p overexpressing cells with NK cells.

High miR‐21‐3p expression levels in both murine and human cells were accompanied by an increased proliferation (Figure S1). BGN^low^ HER‐2/neu^+^ cells constitutively expressed high levels of miR‐21‐3p, while HER‐2/neu^–^ NIH3T3 cells exhibited low miR‐21‐3p expression levels (Figure 1A). BGN overexpression in HER‐2/neu^+^ (BGN^high^/ HER‐2/neu^+^) cells decreased miR‐21‐3p expression (Figure 1A), while the siRNA‐mediated BGN downregulation in NIH3T3 cells increased miR‐21‐3p levels (Figure 1B). Similar results were obtained in vivo demonstrating that miR‐21‐3p expression was higher in BGN^low/neg^ compared to BGN^high^ HER‐2/neu^+^ tumors^3^ (Figure 1C). The inverse correlation of the expression of miRNA‐21‐3p and HER‐2/neu to BGN was confirmed in HTB122 cells expressing functional (E2) or signaling defective (E2A) HER‐2/neu (Figure 1D), but also upon miR‐21‐3p overexpression in NIH3T3 cells (Figure 1E) as well as in the breast cancer (BC) cell line MCF‐7 (Figure 1F), which could be reverted by a miR‐21‐3p inhibitor leading to a statistically significant twofold upregulation of BGN expression.

**FIGURE 1:**
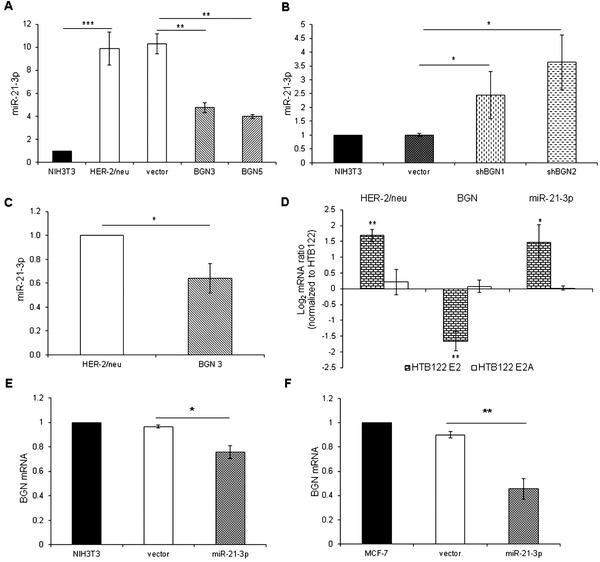
BGN‐mediated reduction of miR‐21‐3p expression in HER‐2/neu^±^ cells. (A) Reduced expression of miR‐21‐3p by BGN overexpression. The constitutive expression of miR‐21‐3p was determined in HER‐2/neu^–^ BGN^high^ NIH3T3 cells, BGN^low^ HER‐2/neu^+^ cells, HER‐2/neu^+^ vector controls as well as two BGN^high^ HER‐2/neu^+^ transfectants by qPCR. The results are presented as x‐fold induction of miR‐21‐3p expression in transfectants compared to parental NIH3T3 cells (set 1). (B) Effect of sh‐mediated BGN silencing on miR‐21‐3p expression in NIH3T3 cells. The BGN expression in NIH3T3 cells was silenced by shRNA and miR‐21‐3p expression was determined by qPCR in shBGN transfectants, vector control, and parental cells. The results are expressed as x‐fold induction of miR‐21‐3p expression in transfectants compared to parental NIH3T3 cells (set 1). (C) Basal miR‐21‐3p expression in BGN^low^ HER‐2/neu^+^ and BGN^high^ HER‐2/neu^+^ tumor cells. The miR‐21‐3p expression in BGN^low^ HER‐2/neu^+^ and BGN^high^ HER‐2/neu^+^ tumor lesions was determined by qPCR analysis and data were presented as x‐fold downregulation of miR‐21‐3p expression. (D) Required HER‐2/neu signaling for miR‐21‐3p upregulation. The expression of HER‐2/neu, BGN, and miR‐21‐3p was determined in HTB122 cells, HTB122 E2, and HTB122 E2A transfectants by qPCR. Data are expressed as log mRNA ratio normalized to parental HTB122 cells. (E) miR‐21‐3p‐mediated effects on BGN expression in NIH3T3 cells. NIH3T3 cells were transfected with the empty vector and miR‐21‐3p, before miR‐21‐3p and BGN expression was determined by qPCR miR‐21‐3p transfection resulted in an x‐fold upregulation of miR‐21‐3p in NIH3T3 cells (set to 1). (F) miR‐21‐3p‐mediated effects on BGN expression in BC cells. MCF7 cells were transfected with miR‐21‐3p and vector control, respectively, before miR‐21‐3p and BGN expression was determined by qPCR. Transfection resulted in a 3.07‐fold upregulation of miR‐21‐3p. Data were normalized to parental MCF7 cells (set to 1)

BGN is modulated by and can modulate the TGF‐β pathway by regulating the expression/activity of SMAD family members.^3^ TGF‐β treatment of the murine in vitro models and human BC cell lines caused a significantly enhanced miR‐21‐3p expression in BGN^low^ HER‐2/neu^+^ cells, a heterogeneous increase in BC cell lines, but only a marginal upregulation in BGN^high^ HER‐2/neu^–^ cells (Figure S2). SMAD2 expression is downregulated in BGN^low^ HER‐2/neu^+^ cells, but significantly enhanced in BGN‐transfected HER‐2/neu^+^ cells (Figure 2A). Vice versa, SMAD2 transfectants of HER‐2/neu^+^ cells caused an increased BGN mRNA expression (Figure 2B), while miR‐21‐3p overexpression leads to a downregulation of SMAD2 (Figure 2C and D). This mechanism was due to the binding of miR‐21‐3p to the 3′ UTR of SMAD2 as demonstrated by in silico analysis,^4^ a high binding energy of miR‐21‐3p to the SMAD2 3′ UTR (Figure 2E) and luciferase (luc) reporter assays (Figure 2F). Next to the effect of miR‐21‐3p on SMAD2 expression in the murine model system, miR‐21‐3p overexpression in the human MCF‐7 cell line downregulated TAP1 expression (Figure 2G), which supported our recent in vitro experiments in melanoma,^5^ the in silico data of miR‐21‐3p binding to the 3′ UTR of human TAP1 (Figure 2H), and the luc reporter assays demonstrating a binding of miR‐21‐3p to the wild‐type TAP1 3′ UTR (Figure 2I).

**FIGURE 2:**
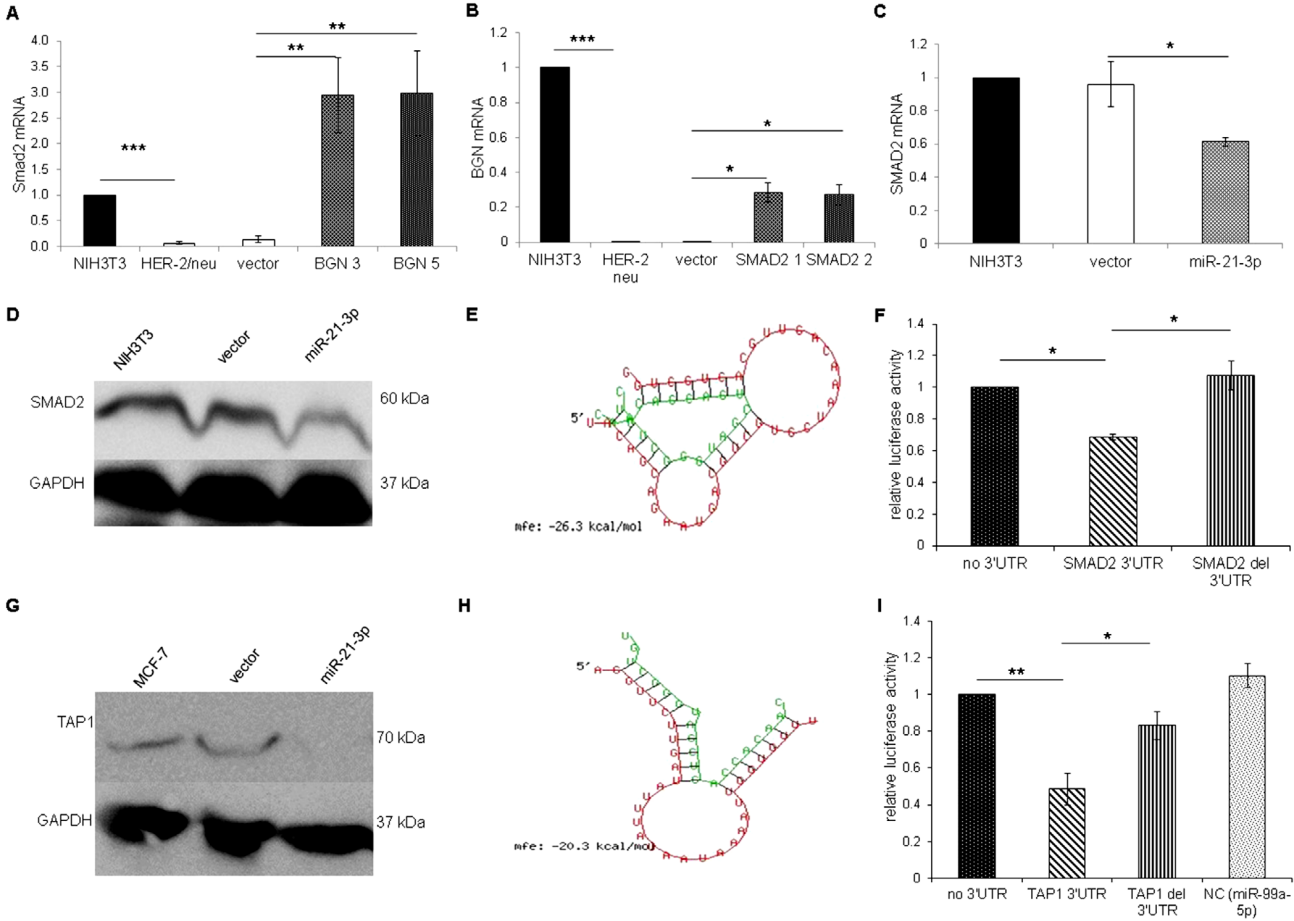
Mouse SMAD2 and human TAP1 as targets of miR‐21‐3p. (A) Correlation of SMAD2 and BGN expression in the murine model system. SMAD2 mRNA expression was determined in HER‐2/neu^–^ cells, BGN^low^ HER‐2/neu^+^ cells and vector controls BGN^high^ HER‐2/neu^+^ using qPCR. Data were normalized to parental NIH3T3 cells (set 1). (B) Restoration of BGN expression in SMAD2 overexpressing HER‐2/neu^+^ cells. BGN expression was analyzed in SMAD2 overexpressing HER‐2/neu^+^ cells by qPCR. Data were normalized to parental NIH3T3 cells set to “1.” (C) Link of SMAD2 with miR‐21‐3p expression. MiR‐21‐3p was transfected into NIH3T3 cells and SMAD2 mRNA expression was determined in miR‐21‐3p transfectants by qPCR. Data were normalized to parental NIH3T3 cells (set 1). (D) Effect of miR‐21‐3p on SMAD2 protein expression SMAD2 protein expression was determined by Western blot analysis of NIH3T3 cells and miR‐21‐3p transfectants using a SMAD2‐specific antibody. An anti‐GAPDH antibody served as loading control. (E) In silico analysis of SMAD2 as a target of miR‐21‐3p. RNA hybrid was used as a tool to determine the binding energy. The high binding energy using RNA hybrid suggested that the 3′ UTR of SMAD2 targets miR‐21‐3p. (F) Direct binding of miR‐21‐3p to the SMAD2 3′ UTR. Luciferase reporter assays were independently performed three times using the wt and del 3′ UTR of SMAD2 and data are expressed as Iuc activities normalized to constructs lacking the 3′ UTR. (G) Reduced TAP1 protein expression upon miR‐21‐3p overexpression. MCF‐7 cells were transfected with vector or miR‐21‐3p, before TAP1 protein expression was determined by Western blot. Staining with an anti‐GAPDH antibody served as control. (H) *In silico* analysis of the 3′ UTR of TAP1. *In silico* analysis using various algorithms postulated that miR‐21‐3p targets the 3′ UTR of human TAP1. RNA hybrid was used as a tool to determine the binding energy of miR‐21‐3p to the 3′ UTR of TAP1. (I) Direct binding of miR‐21‐3p to the 3′UTR of TAP1. Luc activities were determined in a luc reporter assay using wt TAP1 3′ UTR, del TAP1 3′ UTR and a nonsense control. The data are expressed as the mean of luc activity normalized to MCF‐7

Flow cytometric analysis revealed lower MHC class I surface levels in miR‐21‐3p transfected BGN^high^ HER‐2/neu^–^ NIH3T3 cells compared to control transfectants (Figure 3A), while MHC class I surface expression was enhanced in NIH3T3 cells upon transfection with a miR‐21‐3p inhibitor (Figure 3B). Similar results were obtained with human miR‐21‐3p‐transfected MCF‐7 (Figure 3C) and MDA‐MB‐231 (Figure S3A) cells demonstrating downregulated HLA class I surface expression, which was reverted a miR‐21 inhibitor (Figure 3D; Figure S3B). In this context, it is noteworthy that SMAD2 overexpression caused an upregulation of H2‐Lq (Figure S4).

**FIGURE 3:**
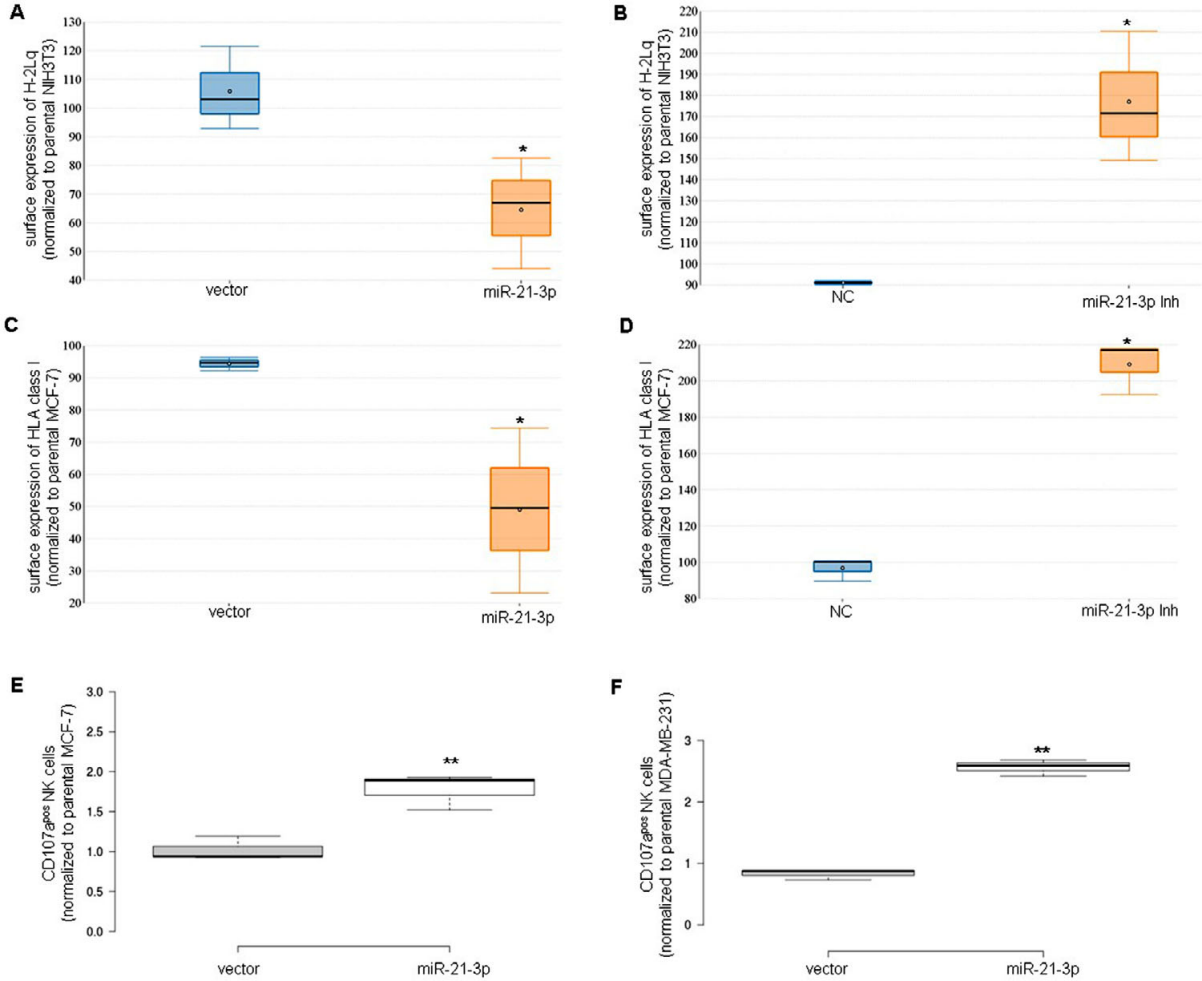
miR‐21‐mediated downregulation of MHC class I antigens and functional relevance. Both murine of NIH3T3 cells and BC cells transfected with miR‐21‐3p or a miR‐21‐3p inhibitor, respectively, were monitored for the expression of MHC class I surface antigens using flow cytometry with MHC class I‐specific antibodies. The results are presented as MFI and normalized to parental cells set to 100. (A) Downregulation of MHC class I surface expression by a miR‐21‐3p transfection of NIH3T3 cells. (B) Upregulation of MHC class I surface expression by a miR‐21‐3p inhibitor transfection of NIH3T3 cells. (C) Reduced HLA class I surface expression in miR‐21‐3p transfected MCF‐7 cells. (D) Reversion of HLA class I surface expression by miR‐21‐3p inhibitor transfection in MCF‐7 cells. (E and F) Functional relevance of miR‐21‐3p‐mediated downregulation of MHC class I. Degranulation assays were performed using NK cells isolated from PBMC obtained from four different donors. Shown are the mean ± standard error of the fold increase in CD107a+ NK cells upon normalization to parental cells, MCF7 (E) and MDA‐MB‐231 (F)

Since the impaired TAP1 expression reduced MHC class I surface expression,^6^ the influence of the miR‐21‐mediated MHC class I downregulation on NK cell responses was determined using the CD107a degranulation assay. As expected, the impaired MHC class I expression of miR‐21‐3p transfectants enhanced NK cell recognition of MCF‐7 (Figure 3E) and MDA‐MB‐231 cells (Figure 3F).

Using The Cancer Genome Atlas (TCGA) data of invasive BC, a link between miR‐21‐3p, HLA class I, and HER‐2/neu expression to clinical parameters was detected. High miR‐21 expression was found in primary BC compared to the normal mammary epithelium (Figure 4A), which correlated to high HER‐2/neu, but low HLA class I expression levels as representatively shown for HLA‐B (Figure 4B) and was accompanied by a worse BC patients’ outcome (Figure 4C).

**FIGURE 4:**
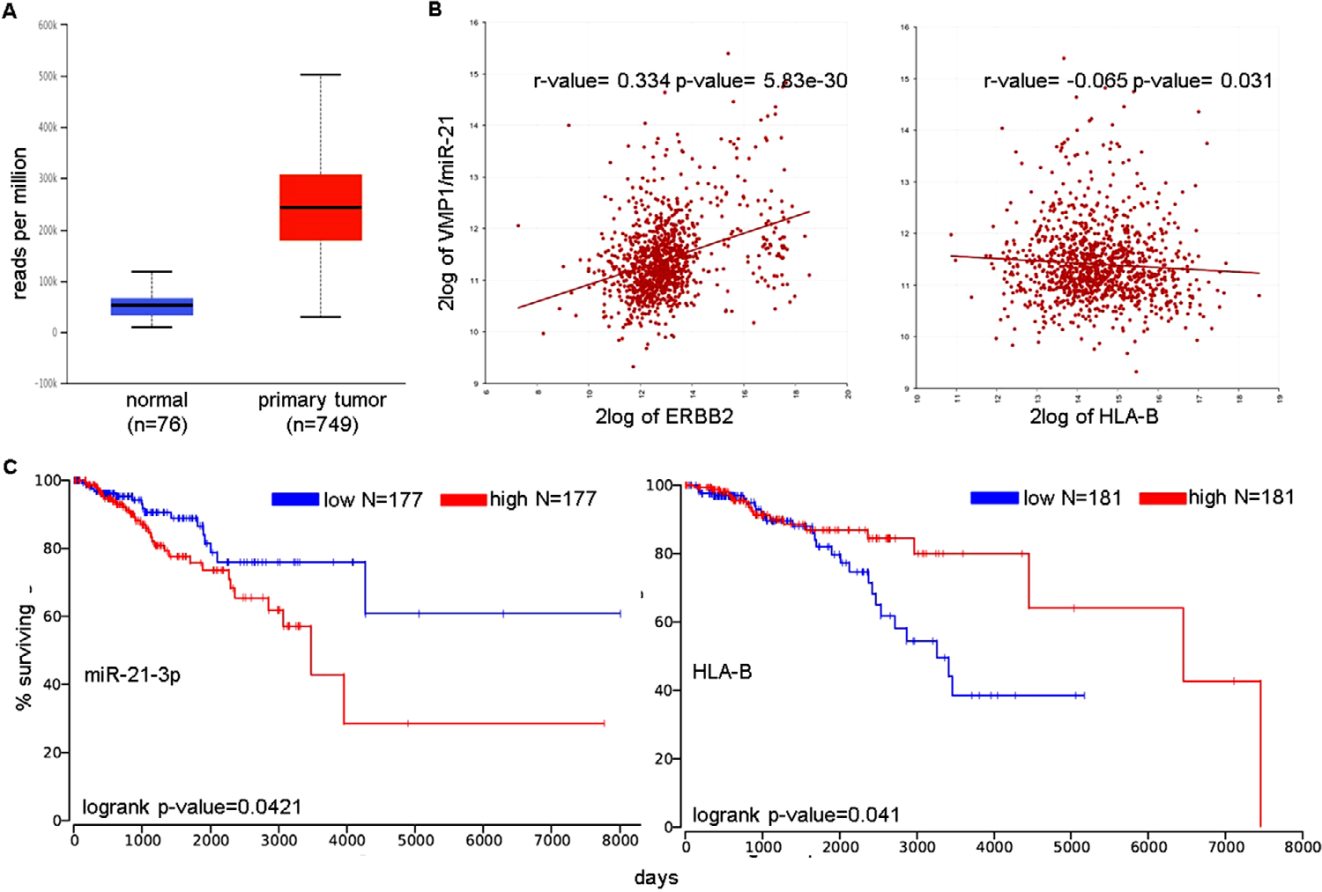
TCGA data analysis of breast cancer samples. (A) miR‐21 expression in normal (*n* = 76) and BC samples (*m* = 749). Box‐whisker plot showing the miR‐21 expression in BC dataset was detected by the UALCAN database. (B) Correlation analysis of the TCGA invasive breast carcinoma dataset (1097 patients) using R2 Genomics was performed between miR‐21 and HER‐2/neu (ERBB2) and HLA‐I genes (representatively shown for HLA‐B). MiR‐21 correlated positively with ERBB2 and negatively with HLA class I antigens. (C) Kaplan Meyer curve demonstrated a higher survival of HLA^high^ and miR‐21‐3p groups as described. The assessment of clinical relevance was performed in a patient survival analysis using OncoLnc database (http://www.oncolnc.org/). While HLA^high^ (HLA‐B) indicated the higher survival, miR‐21‐3p^high^ displayed lower survival for TCGA Breast Invasive carcinoma dataset followed for 20 years

In summary, the murine miR‐21‐3p enhances the TGF‐β signaling by binding to the 3′UTR of SMAD2, resulting in a reduced MHC class I surface expression, while hsa‐miR‐21‐3p binds to the human TAP1 3′UTR thereby restricting the peptide transport and loading of MHC class I antigens (Figure S5A). The BGN‐mediated overexpression in BGN^low/neg^ HER‐2/neu^+^ cells induced MHC class I expression and reduced miR‐21‐3p (Figure S5B), underlining its critical role on the MHC class I‐mediated tumor immune escape. The crosstalk between HER‐2/neu and miR‐21‐3p alters the intracellular signaling in cancer cells by promoting cell proliferation, enhancing pro‐invasive growth factors, like TGF‐β and inhibiting immune stimulatory molecules or ECM components, like BGN.

These data postulate the pharmacological targeting of the miRNA‐proteoglycan‐MHC class I axis as a novel, innovative therapeutic concept for HER‐2/neu^+^ cancers.

## CONFLICTS OF INTEREST

All authors declare that there is disclose competing interest.

## FUNDING INFORMATION

Wilhelm‐Sander‐Stiftung, Grant Number: 2019.076.1; Mildred Scheel Stiftung; Grant Number: 70113861.

## DATA AVAILABILITY STATEMENT

The authors will make the data and material used available upon request.

## Supporting information

Supporting Information
