# Supplementary material for: Identification of a novel miR‐21‐3p/TGF‐β signaling‐driven immune escape via the MHC class I/biglycan axis in tumor cells
Source: Clin Transl Med. 2021 Mar 24;11(3):e306. doi: 10.1002/ctm2.306 (PMC7989704; doi:10.1002/ctm2.306)
Supplement: Supplementary file 1 — Supporting Information [file CTM2-11-e306-s001.pdf]

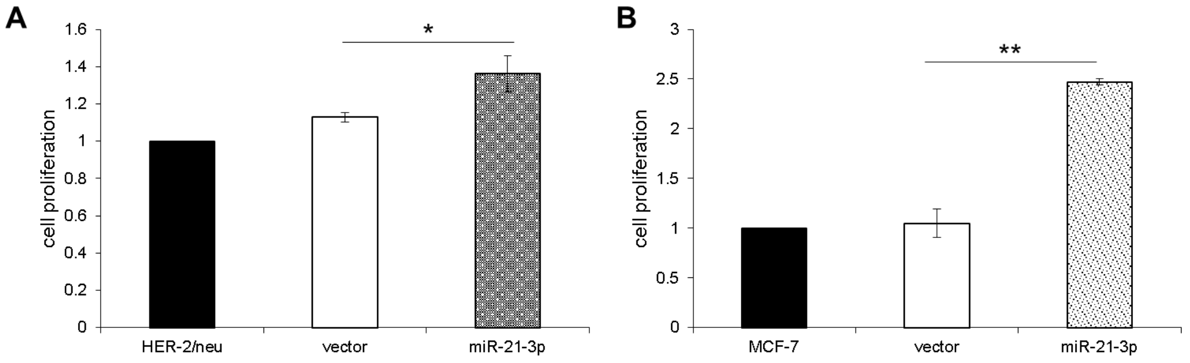

<u>Supplementary Figure 1:</u> Enhanced cell proliferation of HER-2/neu (A) and MCF (B) cells upon transfection with the miR-21-3p inhibitor.

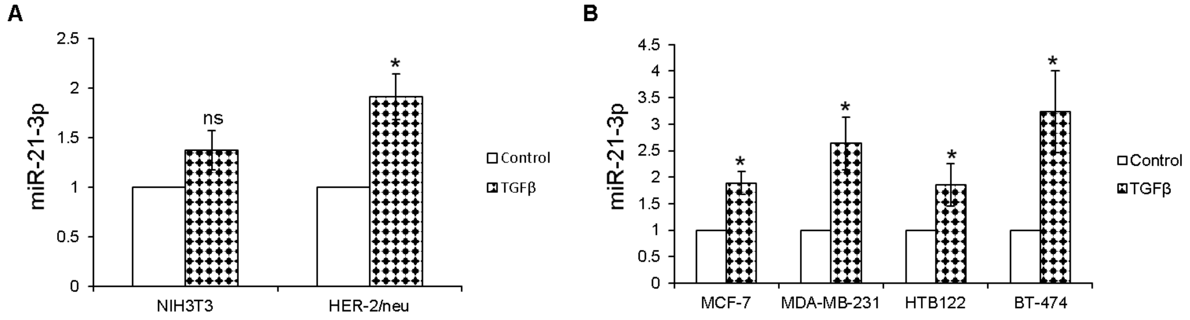

<u>Supplementary Figure 2: Upregulation of miR-21-3p in murine and human cell model systems upon TGF-β treatment.</u>

A: NIH3T3 cells and BGN^{low} HER-2/neu^{+} cells were treated for 48 hr with TGF-β and miR-21-3p expression was determined by qPCR. The data are expressed as x-fold induction of miR21-3p to untreated cells (set 1).

B: Upregulation of miR-21-3p in human tumor cells by TGF-β. Four human BC cell lines were treated for 48 hrs. with TGF-β and miR-21-3p was determined by qPCR. The data are expressed as x-fold induction of miR-21-3p to untreated cells (set to 1).

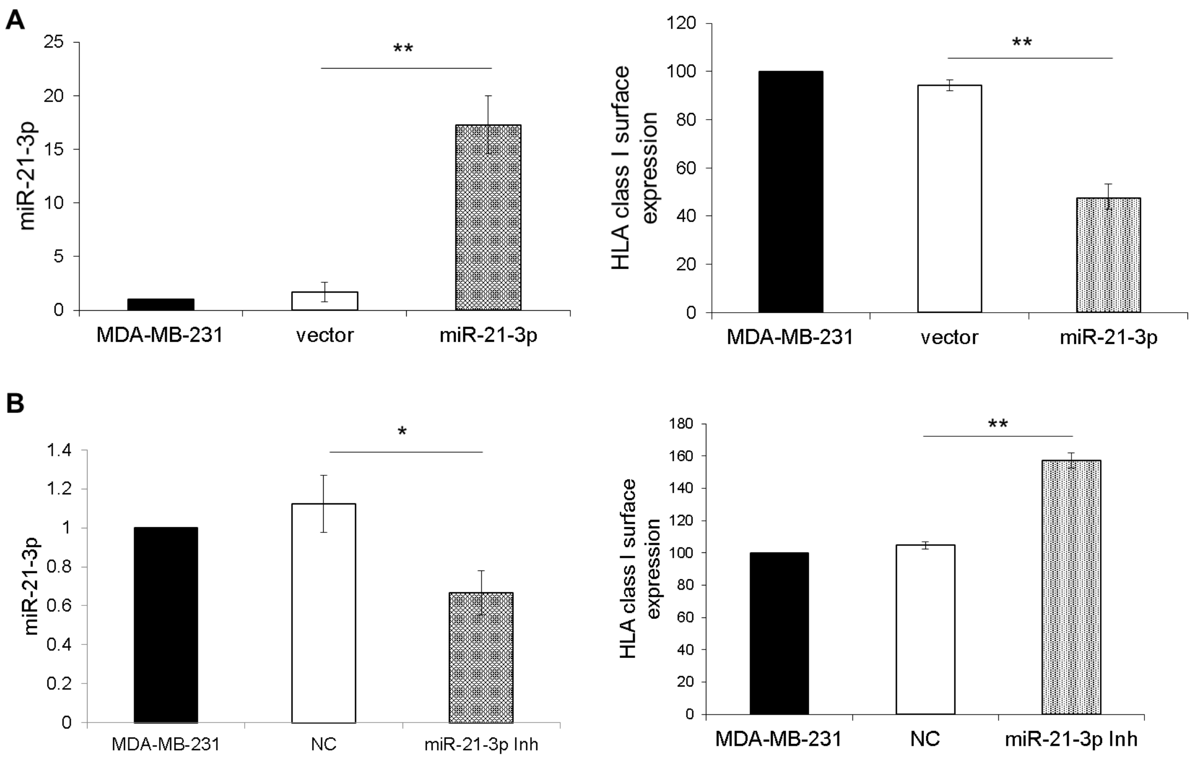

Supplementary Figure 3: miR-21-mediated downregulation of MHC class I antigens in MDA-MB-231 cells

MDA-MB-231 cells transfected with miR-21-3p were monitored for the expression of HLA class I surface antigens by flow cytometry using HLA class I specific antibodies. The results are presented as MFI and correlated to parental cells set to 100.

A: Reduced HLA class I surface expression in miR-21-3p transfected MDA-MB-231 cells.

B: Reversion of HLA class I surface expression by miR-21-3p inhibitors in MDA-MB-231 cells.

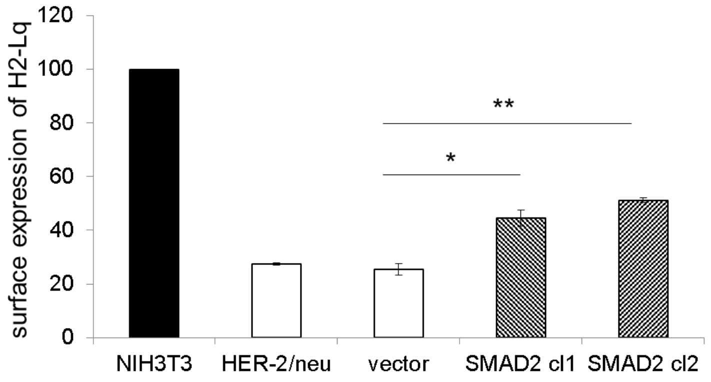

Supplementary Figure 4: Induction of MHC class I expression upon SMAD2 overexpression in HER-2/neu+ cells MHC class I surface expression was performed by flow cytometry. The data are presented as MFI.

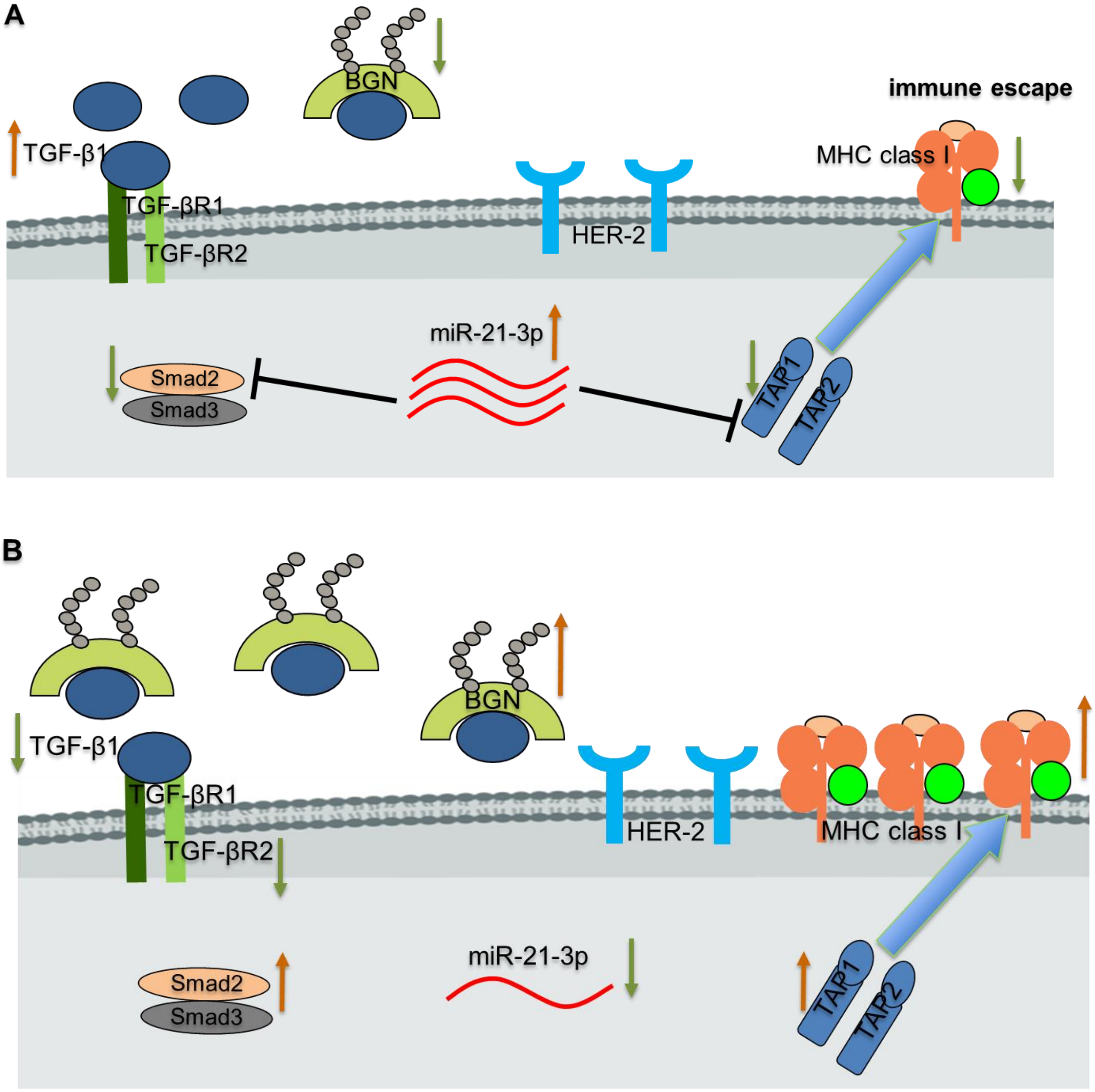

<u>Supplementary Figure 5: Schematic representations</u>

A: Underlying mechanisms of the miR-21-3p-mediated downregulation of MHC class I in HER-2/neu model systems.

B: BGN-mediated overexpression induced MHC class I expression and reduced miR-21-3p via restricted TGF-β signaling.

Supplementary Table 1: Real-time quantitative PCR primers

Gene	Forward	Reverse
**Mouse**		
BGN	GGACTCTGTCACACCCACCT	CTTGTTGTTCACCAGGACGA
SMAD2	ATGTCGTCCATCTTGCCATTC	AACCGTCCTGTTTTCTTTAGCTT
GAPDH	TCTGCTGGAAGGTGGACAGT	CCTCTATGCCAACACAGTGC
β-action	AAGGTCATCCCAGAGCTGAA	CTGCTTCACCACCTTCTTGA
miR-21-3p	ACACTCCAGCTGGGCAACAGCAGTCGATGG	CTCAAGTGTCGTGGAGTCGGCAA
U6	GCTTCGGCAGCACATATACTAAAAT	CGCTTCACGAATTTGCGTGTCAT
	Stem-loop reverse transcription (SLRT) primers	
miR-21-3p	CTCAACTGGTGTCGTGGAGTCGGCAATTCAGTTGAGGACAGCCC	
U6	CGCTTCACGAATTTGCGTGTCAT	
**Human**		
BGN	ACCTCCCTGAGACCCTGAAT	CTGGAGGAGCTTGAGGTCTG
HER-2/neu	CCAGCAGGGCTTCTTCTGT	TCCAGCCCTAGTGTCAGGTC
TAP1	GGAATCTCTGGCAAAGTCCA	TGGGTGAACTGCATCTGGTA
GAPDH	CTGGTAAAGTGGATATTGTTGCCAT	TGGAATCATATTGGAACATGTAAACC
β-action	ACTCTTCCAGCCTTCCTTCC	AGCACTGTGTTGGCGTACAG
miR-21-3p	CGCGCCAACACCAGTCGATG	GTGCAGGGTCCGAGGT
U6	CTCGCTTCGGCAGCACA	AACGCTTCACGAATTTGCGT
TAP1 3’UTR LUC	AAAGCTAGCCTCCAGAATGAAAGCCTTCTC	AAAGTCGACACAAAACACCAATTTTATTA
del TAP1 3’UTR miR-21-3p	GTGTCGACCTGCAGGCAT	ACAGGGTGTTTATGGGCC
	Stem-loop reverse transcription (SLRT) primers - miR-21-3p	
miR-21-3p	GTCGTATCCAGTGCAGGGTCCGAGGTATTCGCACTGGATACGACACAGCC	
